# The Prevalence of Epilepsy Among Patients With Alzheimer’s Disease at the King Abdulaziz Medical City, Riyadh, Saudi Arabia

**DOI:** 10.7759/cureus.89474

**Published:** 2025-08-06

**Authors:** Hesham N Alotaibi, Fawaz Al-Ibrahim, Yazeed N Aljuhami, Hisham A Alshahrani, Naif M Alzahrani, Turki Alnemer, Nasser Alsahani, Moayad Y Alamoudi, Aamir Omair, Talal Aljumah

**Affiliations:** 1 Medicine, King Saud Bin Abdulaziz University for Health Sciences, Riyadh, SAU; 2 Research, King Abdullah International Medical Research Center, Riyadh, SAU; 3 Neurology, King Abdulaziz Medical City, Riyadh, SAU; 4 Medicine, Imam Abdulrahman Bin Faisal University, Dammam, SAU; 5 Research, King Abdulaziz Medical City, Riyadh, SAU; 6 Medical Education, King Saud Bin Abdulaziz University for Health Sciences, Riyadh, SAU

**Keywords:** alzheimer's disease, cognitive decline, epilepsy, epileptic seizures, prevalence, saudi arabia

## Abstract

Background

Alzheimer’s disease (AD) and epilepsy are neurological conditions that can affect elderly individuals. The coexistence of these two conditions may lead to additional health challenges and negatively impact patient outcomes. The study aimed to assess the prevalence of epilepsy among patients with AD in Saudi Arabia and to explore factors associated with its occurrence.

Methods

A cross-sectional retrospective study was conducted at King Abdulaziz Medical City, Riyadh, reviewing the records of patients with AD from 2016 to 2022. Patients with prior epilepsy or without a brain MRI were excluded. Data on demographics, comorbidities, medications, and imaging were analyzed.

Results

Among 385 patients (mean age 75.5 years) included in the study, epilepsy prevalence was 6%. Diabetes (82%), hypertension (78%), and hyperlipidemia (63%) were common but showed no significant association with epilepsy. Generalized brain atrophy was the most frequent MRI finding. Levetiracetam was the most prescribed antiepileptic drug (77%).

Conclusion

Epilepsy is more common among patients with AD than in the general population. Early recognition and management are crucial. Further research is needed to understand the risk factors and improve outcomes.

## Introduction

Neurological disorders such as Alzheimer's disease (AD) and epilepsy are commonly seen in the elderly population. AD is a neurodegenerative disease that can cause severe memory loss in late stages, which is a type of dementia [1]. In 2005, about 24 million people were living with dementia, and it was estimated that 4.6 million cases occurred annually [2]. Epilepsy is a brain disorder characterized by an enduring predisposition to generate epileptic seizures [3]. Patients with AD are six times more likely to suffer seizures and epilepsy [4].

In the general population, the prevalence of epilepsy is between four to 12 per 1000 adults, and in Saudi Arabia, the prevalence is 6.5 per 1,000 individuals [3,5]. In Arab countries, research shows that the prevalence of dementia ranges from 1.1%-2.3% in people aged 50 years and older [6]. On the other hand, the prevalence of epilepsy in patients with AD varied between 10% and 64% across different epidemiological studies in different countries and population groups [7,8]. This inter-study variability may be due to the complexity and difficulty of estimating seizures in patients with dementia, and fluctuations in levels of consciousness may be due to focal seizures [7]. Moreover, the challenge of recalling unusual episodes, especially focal seizures, by caregivers was difficult [7]. However, using antiepileptic drugs to treat seizures may be highly effective in slowing cognitive decline in people with AD [9]. The choice of a specific antiepileptic drug is individualized based on drug interactions, comorbidities, and each patient's tolerance of other drugs [3]. Research by Sen et al. shows that epilepsy can increase the risk of developing of AD [10]. This indicates some association between the two conditions [7]. Both conditions have similar risk factors such as obesity, diabetes, hypertension, and smoking [10].

Research has shown that seizures can speed up cognitive decline in patients with AD [8]. It has been shown that patients with temporal lobe epilepsy have accelerated long-term memory decline [10]. Patients in the pre-symptomatic stage of AD who had epilepsy showed impairment 3.6 years sooner in comparison to patients who had no history of epilepsy [11]. Additional evidence also supports an increased risk of epilepsy in patients with AD [8,12].

This study is important because there is limited research discussing the prevalence of epilepsy in patients with AD in Saudi Arabia and other countries. It highlights an unrecognized problem, both locally and globally. It also focuses on the risk factors of developing epilepsy in patients with AD. The aim was to identify the prevalence of epilepsy among patients with AD and explore the associated risk factors, treatment patterns, imaging findings, and comorbidities.

## Materials and methods

This study was conducted in the Neurology Division of King Abdulaziz Medical City (KAMC) in Riyadh, Saudi Arabia. KAMC-Riyadh provides all types of treatment from primary care to tertiary facilities. The neurological division has 16 consultants, and they treat different types of disorders, e.g., stroke, epilepsy, AD, etc. This was a cross-sectional study aimed at determining the prevalence of epilepsy in patients with AD.

The research group collected the data on patients with AD admitted to KAMC from 2016 to 2022 retrospectively. All patients who had been diagnosed with AD were included in the study. However, those who were previously diagnosed with epilepsy before AD or who did not have a brain MRI study were excluded. Non-probability sampling was used, and data were collected on all patients who met the inclusion criteria. AD was diagnosed based on the clinical criteria from the National Institute on Aging and the Alzheimer’s Association (NIA-AA) [13]. These criteria include memory decline along with impairment in at least one other cognitive domain, as well as a noticeable effect on daily functioning. Cognitive tests like the Mini-Mental State Examination (MMSE) or Montreal Cognitive Assessment (MoCA) were used when available. Other causes of dementia (such as B12 deficiency or thyroid issues) were excluded. In some cases, neuroimaging (MRI or CT) or biomarkers (like cerebrospinal fluid (CSF) tau or amyloid, or amyloid positron emission tomography (PET)) were also used to support the diagnosis if the data were available. Epilepsy was diagnosed according to the International League Against Epilepsy (ILAE) criteria. This includes either two or more unprovoked seizures more than 24 hours apart, or one unprovoked seizure with a high risk (≥60%) of recurrence. Some patients were also diagnosed based on clear epilepsy syndromes. Diagnosis was based on the clinical history, EEG findings, and brain imaging (like MRI) [14]. The Raosoft calculator was used to determine the required sample size. By using a confidence level of 95% with a prevalence of 10% and a margin of error of 3%, the required sample size was estimated to be 377 [8,15].

Data were extracted from the BestCare system (the electronic medical records system of KAMC, Riyadh) using the patients’ medical record numbers (MRN) from the King Abdullah International Medical Research Center. The data were entered in an Excel sheet (Microsoft Corp., Redmond, WA, US). The data sheet included baseline characteristics of the study population, such as age and gender as well as comorbidities such as hypertension, diabetes mellitus, end-stage renal disease, and chronic liver disease. The following variables were also collected for all patients: date of AD diagnosis and the medications used (e.g., mementine, rivastigmine). The main outcome variable was the epilepsy status, and the main grouping variables were the baseline characteristics like gender and age.

All the data were exported to IBM SPSS Statistics for Windows, Version 27 (Released 2020; IBM Corp., Armonk, New York, United States) to be analyzed. Categorical data (e.g., epileptic status, gender) were presented as frequency and percentages. Numerical data (e.g., age, duration of diagnosis) were presented as mean and standard deviation. Chi-square test and Fisher's exact test were used to compare epileptic and non-epileptic patients by gender, age group and other variables. A test was considered to show a significant association if the p-value was less than 0.05.

All the patient information was protected and only seen by the research group. Consent was not required since the research team reviewed the patient charts. Institutional Review Board (IRB) approval was obtained from King Abdullah International Medical Research Centre (approval no. IRB/1778/22). To ensure the patient confidentiality, all personal identifiers were removed from the records, e.g., name, medical record number, and ID number.

## Results

A total of 385 valid observations were recorded for both age and age at diagnosis, with no missing data. The mean age of the participants was 75.5 years (SD=9.2), and the mean age at diagnosis was 74.9 years (SD=9.1). The ages ranged from 23 to 99 years for both variables.

All 385 patients were diagnosed with one or more comorbidities (Table 1).

**Table 1:** Comorbidities in patients with Alzheimer’s disease (n=385) NAFLD: Non-alcoholic fatty liver disease; Data presented as frequencies and percentages.

Condition	Number of patients (n)	Proportion (%)
Diabetes	315	82%
Hypertension	300	78%
Hyperlipidaemia	241	63%
Stroke	117	30%
Arthritis	81	21%
Renal Disease	73	19%
Depression	70	18%
Heart Failure	64	17%
Cancer	32	8%
Asthma	27	7%
NAFLD	8	2%

The most common comorbidity was diabetes (n=315; 82%), followed by hypertension (n=300; 78%) and hyperlipidemia (n=241; 63%). The three least common comorbidities were cancer (n=32; 8%), asthma (n=27; 7%), and non-alcoholic fatty liver disease (NAFLD; n=8; 2%).

Table 2 shows the number of drugs and their usage among patients with AD diagnosed with epilepsy (n=22, 6%).

**Table 2:** Epilepsy medications used by patients with Alzheimer's disease and epilepsy (n=22) Note: Some patients were prescribed more than one medication; Data presented as frequencies and percentages.

Medication	Number of patients (n)	Proportion (%)
Topiramate	1	5%
Carbamazepine	2	9%
Clonazepam	1	5%
lamotrigine	3	14%
Phenytoin	5	23%
Valproate (Valproic acid)	4	18%
Pregabalin	3	14%
Levetiracetam	17	77%

Among these 22 patients, some were on more than one medication. The results showed that levetiracetam was the most commonly used medication (n=17; 77%) patients, followed by phenytoin (n=5; 23%) and valproate (n=4; 18%). 

**Figure 1:**
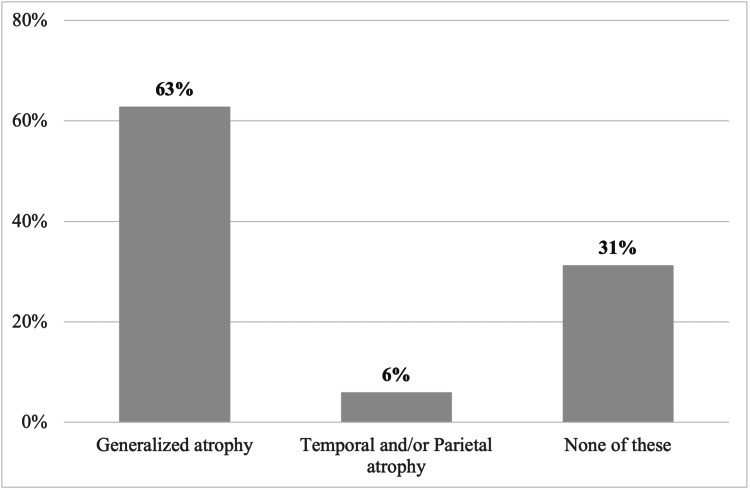
Results of the brain MRI in patients with Alzheimer's disease (n=385)

The results from the brain MRI (n=385) were categorized into three outcomes, as shown in Figure 1.

The majority of patients had generalized cortical atrophy (n=243; 63%). A minority, showed either temporal or parietal atrophy, or both (n=21; 6%). The last set of patients showed neither generalized nor temporal/parietal atrophy (n=121; 31%).

Table 3 shows the comorbidities in patients diagnosed with epilepsy compared to those without it.

**Table 3:** Prevalence of comorbidities in patients diagnosed with or without epilepsy *Percentages are given as column percentages out of those with epilepsy diagnosis or not ^a^Fisher's exact test; p-value significant at p<0.05

	Epilepsy diagnosis					
	Yes (n=22)		No (n=363)			
	n	%*	n	%*	Chi-Square value	p-value^a^
Arthritis	2	9%	79	22%	2.005	0.24
Asthma	2	9%	25	7%	0.155	0.93
Cancer	0	0%	32	9%	2.115	0.28
Depression	6	27%	64	18%	1.296	0.38
Diabetes	18	82%	297	82%	0	>0.999
Heart failure	4	18%	60	17%	0.04	>0.999
Hyperlipidemia	11	50%	230	63%	1.582	0.30
Hypertension	18	82%	282	78%	0.206	0.89
Non-alcoholic fatty liver disease	1	5%	7	2%	0.698	0.76
Renal disease	5	23%	68	19%	0.215	0.81
Stroke	9	41%	108	30%	1.221	0.38

The prevalence of diabetes was similar in the group with epilepsy (n=18; 82%) and without (n=297; 82%), with no significant difference between the groups (p>0.999). This was true for hypertension too, with a comparable prevalence in those with epilepsy (n=18; 82%) and without epilepsy (n=282; 78%), showing no significant association (p=0.89). Based on the p-values from Fisher’s exact test, none of the comorbidities showed a significant difference between the patients diagnosed and not diagnosed with epilepsy.

Generalized cortical atrophy was observed in 11 patients with epilepsy (50%) and in 232 patients without epilepsy (64%). Temporal, parietal, or combined atrophy was noted in three patients with epilepsy (14%) and in one patient without epilepsy (5%). Regarding EEG findings, generalized slowing was observed in eight patients with epilepsy (53%) and 34 patients without epilepsy (37%). Normal EEG results were reported in four patients with epilepsy (27%) and 35 patients with epilepsy (38%). Focal EEG abnormalities (including right, left, or temporal slowing) were identified in one patient with epilepsy (7%) and nine patients without epilepsy (9%). Based on the p-value, there was no statistically significant difference in the prevalence of specific brain MRI findings or EEG results between patients with or without epilepsy (Table 4).

**Table 4:** Comparison of brain MRI and EEG results between patients diagnosed with and without epilepsy ^*^Percentages are given as column percentages out of those having epilepsy diagnosis or not; ^a^Fisher's exact test; ^b^Chi-Square test; p-value significant at p<0.05 Note: 106 patients had available EEG results

		Diagnosis of epilepsy					
		Yes (n=22)		No (n=363)			
		n	%*	n	%*	Chi-Square value	p-value
Brain MRI result	None of these	8	36%	113	31%	3.681	0.16^b^
	Temporal or parietal atrophy or both	3	14%	18	5%		
	Generalized atrophy (loss of volume)	11	50%	232	64%		
		Yes (n=15)		No (n=91)			
EEG result	Normal	4	27%	35	38%	2.563	0.74^a^
	Epileptiform discharges and spikes	0	0%	5	5%		
	Focal (Right, Left, Temporal, Slowing)	1	7%	9	10%		
	Generalized slowing	8	53%	34	37%		
	Other	2	13%	8	9%		

## Discussion

The study showed that 6% of patients with AD developed epilepsy. This is consistent with a previous study by Pandis et al., which found that epilepsy prevalence in patients with AD was between 6% and 9.8%, depending on the disease severity [7]. However, a study conducted in Minnesota reported that the prevalence of epilepsy in the general elderly population is approximately 1.7% [16]. These findings support the conclusion that epilepsy is more common in patients with AD than in the general population.

Levetiracetam was found to be the most used antiepileptic medication (n=17) in the group of patients with AD and epilepsy. It is broad-spectrum antiepileptic that is effective against generalized myoclonic seizures, focal seizures, and generalized tonic-clonic seizures [17]. In a study done by Scheltens et al., the MRI consistently revealed marked atrophy of the medial temporal lobes, particularly in the hippocampus formation, in a patient with probable AD [18]. 

The analysis found that among patients with AD and epilepsy, the most common comorbidities were diabetes (82%), hypertension (82%), and hyperlipidemia (50%). These numbers were similar in patients with AD but without epilepsy, showing no significant association between the comorbidities and the occurrence of epilepsy. This might be due to the relatively higher mean age of patients in our study, 75.5 (SD=9.2) years, along with the prevalence of diabetes mellitus and hypertension in the Saudi population, which were 39.5% and 9.2% respectively [19,20]. In general, patients with epilepsy present with medical and psychiatric comorbidities, which may impact the disease outcome. In a study, 18% of individuals with epilepsy had psychiatric comorbidities, while 5.7% had cardiac conditions [21]. 

In a study conducted in Riyadh on 214 patients who were diagnosed with AD, 19 of them had unprovoked seizures [22], but these patients were not diagnosed with epilepsy. It indicated that both hypertension and autoimmune disease can increase seizure risk in patients with AD. The study also showed that patients with severe AD have an increased risk of developing seizures [22].

The prevalence of epilepsy among our sample was 6%. This is in contrast to the general population globally, where the epilepsy prevalence is 0.638% [23]. The increased prevalence might be caused by a high rate of comorbidities such as stroke (41% in patients with epilepsy vs. 30% in those without epilepsy). Further studies are needed to investigate the causative factors. Given the increased prevalence of epilepsy in AD, clinicians should be vigilant in monitoring for seizure activity in patients with AD, particularly in those with temporal or parietal atrophy. The high burden of comorbidities like diabetes and hypertension also underscores the need for comprehensive risk management in these patients to potentially reduce the risk of seizures. Moreover, mental decline could be faster in patients with epilepsy, and treating them with anticonvulsants might slow the progression and decline [9].

This was a single-center study conducted in a Neurology division of a hospital in Riyadh, limiting its generalizability. Due to the small number of epilepsy cases identified, performing multivariate analysis to control for confounding factors was not feasible, and this represents a limitation of the current study. Additionally, as a retrospective study, undiagnosed cases of epilepsy could have been missed. The AD diagnosis of the patients was provided by the King Abdullah International Medical Research Center, based on the International Classification of Diseases (ICD) code which could have led to missed cases. The duration from diagnosis of AD to seizure onset and classification of dementia severity were not consistently available from the retrospective chart review. Future prospective studies with larger samples and longitudinal follow-up could provide a more detailed understanding of epilepsy progression in AD.

## Conclusions

The study provides insight into the prevalence of epilepsy among patients with AD, highlighting the connection between these two neurological conditions. The findings suggest that epilepsy is more common in patients with AD than in the general population, emphasizing the need for awareness and proper management. While various factors were examined, including comorbidities, brain imaging, and treatment approaches, no definitive associations were established. The study also highlights the challenges in diagnosing epilepsy in patients with AD due to overlapping symptoms and cognitive decline. Given these complications, further research is necessary to better understand the relationship between AD and epilepsy, improve early detection, and enhance treatment strategies. Future studies with larger sample sizes and different methodologies could provide more conclusive evidence and contribute to better outcomes.
